# Development of a co‐designed, evidence‐based, multi‐pronged strategy to support normal birth.

**DOI:** 10.1111/ajo.13529

**Published:** 2022-04-13

**Authors:** Jyai Allen, Jocelyn Toohill, Debra K. Creedy, Emily J. Callander, Jenny Gamble

**Affiliations:** ^1^ Transforming Maternity Care Collaborative Brisbane Queensland Australia; ^2^ School of Nursing and Midwifery Griffith University Brisbane Queensland Australia; ^3^ Office of Chief Nurse and Midwifery Officer Clinical Excellence Queensland, Queensland Health Brisbane Queensland Australia; ^4^ Monash Centre for Health Research and Implementation, School of Public Health and Preventive Medicine Monash University Melbourne Victoria Australia; ^5^ School of Nursing, Midwifery and Health Coventry University Coventry UK

**Keywords:** caesarean section, maternity, pregnancy, birth, women, health services

## Abstract

Australia's caesarean section (CS) rate has been steadily increasing for decades. In response to this, we co‐designed an evidence‐based, multi‐pronged strategy to increase the normal birth rate in Queensland and reduce the need for CS. We conducted three workshops with a multi‐stakeholder group to identify a broad range of options to reduce CS, prioritise these options, and achieve consensus on a final strategy. The strategy comprised of: universal access to midwifery continuity‐of‐care and choice of place of birth; multi‐disciplinary normal birth education; resources to facilitate informed decision‐making; respectful maternity care and positive workplace culture; and establishment of a Normal Birth Collaborative.

## INTRODUCTION

Caesarean section (CS) can be lifesaving for mothers and babies. However, the global rise in CS is ‘unprecedented and unjustified’.^1^ The Federation of International Gynaecologists and Obstetricians calls for urgent action to stop unnecessary CSs and address the ‘caesarean section epidemic’.^2^ Australia's CS rate has doubled over the past three decades, from 17.5% in 1990^3^ to 35.0% in 2018.^4^ The potential for negative health impacts from possible overuse of CS are accentuated by the substantial proportion of CSs that are performed on women of less than 39 weeks gestation in the absence of a medical or obstetric indication.^5^ Approximately 37% of women who give birth for the first time in Australia will experience a CS.^4^ Even for women at lowest risk of complications (mother aged 20–34 years, singleton pregnancy, vertex presentation, giving birth at 37–41 weeks gestation), CS rates for first births have risen from 25.3% in 2004 to 30.1% in 2018.^6^ This high rate of CS in first time mothers is of concern, as the rate of successful vaginal birth after CS (VBAC) in Australia remains low and declining (13.1% in 2007 to 12.1% in 2018).^6^

The rate of CS in the Australian state of Queensland is higher than the international average in Organisation for Economic Co‐operation and Development countries, but similar to the rest of Australia. In 2018, 35.2% of all women in Queensland had a CS (planned 12.6%, unplanned 22.6%).^7^ In Queensland public hospitals, 26.7% of women having their first baby had a CS. Only 20% of women had a VBAC.^8^ For women giving birth without a prior CS (first birth or previous vaginal birth), 17% had a primary CS in Queensland public hospitals.^9^ Women giving birth in Queensland also have some baseline differences compared to all Australian women giving birth. Notably, women in Queensland are more likely to be Indigenous (6% vs 4%), have a pre‐existing medical condition (25% vs 19%) and live outside a major city (50% vs 29%).^10^ Older maternal age and obesity are associated with CS. In Queensland, approximately 20% of women giving birth are aged ≥35 years and 20% have a ≥30 kg/m^2^ body mass index.^5^

The *Queensland Clinical Guideline: Normal Birth,* ^11^ supports clinicians in the provision of care to promote normal birth. While this is useful for guiding clinical practice, it does not provide strategic guidance at the service, system, or policy level. New South Wales developed a policy^12^ that sought to increase vaginal births and decrease CS rates; however, this was later reviewed and withdrawn. Internationally, the United Kingdom's Better Births,^13^ and Canada's Joint Policy Statement on Normal Births^14^ are examples of policies that provide guidance at the service, system or policy level. No such policy currently exists within Queensland. There is a growing evidence base for opportunities to reduce the use of CS without compromising maternal, fetal, or neonatal morbidity and mortality.

Considering the high rates of CS in Queensland, including cumulative impact of repeat CS following primary CS, we undertook a project aiming to co‐design an evidence‐based, multi‐pronged strategy (the Strategy) to increase the normal birth rate in Queensland public hospitals. The project objectives were to:

- analyse the drivers of CS in Queensland
- facilitate a multi‐stakeholder group to co‐design a coherent strategy to increase normal birth
- prioritise key activities, identify enablers and resisters, and rate the degree of complexity and difficulty of each activity
- determine who is needed to fully design, implement, and evaluate the key activities.

## CO‐DESIGN APPROACH

To innovate, design and create the Strategy we used a co‐design methodology to facilitate a collaborative iterative staged process, with multi‐disciplinary key stakeholders who have extensive experience in maternity care in Queensland.^15^

Key stakeholders from across Queensland Health were invited to participate in a series of three online workshops conducted between March and June 2021. Sixteen participants contributed to each 90‐min workshop. Participants included six obstetricians (one maternal fetal medicine, five tertiary obstetrics), one rural and remote medicine specialist, five midwives, two First Peoples midwives, and two consumer representatives. The workshops were video‐recorded, and a senior researcher took detailed notes. The notes were summarised, checked for accuracy, and performed content analysis analysed after each workshop to develop themes. Some participants emailed additional ideas and information that were collated and analysed. Findings from the previous workshop were presented for participants to consider in advance of the subsequent workshop. Prior to the final workshop, participants had an opportunity to review and provide feedback on the draft Strategy.

### Workshop 1

Prior to the workshop, participants were emailed a desktop literature review of key drivers of CS in Queensland (Box 1). These included clinical, political/policy, economic, socio‐cultural, technological, legal and environmental drivers. Objectives of the first workshop were to canvass aspirational ideas about activities to reduce CS in Queensland that addressed key drivers (Box 2). Following the workshop, participants were invited to discuss their ideas more broadly within their workplaces and with their colleagues to refine them. Participants identified three^3^ priority areas to address safe reduction of CS:

- redesign maternity care
- institutional enablers
- workforce capability.

Box 1 **Drivers of caesarean section**

1. **Clinical**
  High rates of induction of labour and epidural analgesia, along with low rates of VBAC directly, or indirectly, drive CS.
2. **Political and policy**
  There is no national policy or campaign to address overuse of CS. The *Queensland Normal Birth Clinical Guideline* is not well‐integrated with other policies and initiatives.
3. **Economic**
  Activity‐based funding provides a financial disincentive to reduce CS.
4. **Socio‐cultural**
  Medical model and fear of birth undermines clinician and community confidence and devalues normal birth.
5. **Technological**
  Maternity services in Queensland have invested heavily in medical technologies which directly, or indirectly, drive CS rates without delivering clinical benefit.
6. **Legal**
  Fear of medico‐legal consequences for not performing CS lowers obstetric threshold which drives up rates of CS.
7. **Environmental**
  Birth suite environment increases risk of CS compared to birth centre and homebirth for women at low risk of complications.

Box 2 **List of activities identified by participants, grouped by priority areas.**

**1. Redesign maternity care**

*Redesign midwifery care*

Provide universal access to a best‐practice model of midwifery continuity of care

Provide access to Birthing on Country for First Peoples women

Provide community‐based midwifery antenatal and postnatal visits

*Redesign obstetric care*

Remove routine obstetric appointments for women with normal pregnancies

Provide continuity of obstetrician / registrar for antenatal care

Provide multi‐disciplinary obstetric visits close to when issue identified

*Redesign birth setting*

Provide publicly funded homebirth and access to homebirth

Increase access to birth centres

Use birth unit design principles to facilitate normalisation in birth suite

**2. Institutional barriers and enablers**

*Remove financial barriers to preventative, quality maternity care*

Queensland Health provide health services with bundled funding for each woman's pregnancy/birth/postpartum care

Funding for one‐to‐one ratios for midwives caring for women in labour on birth suite

*Use outcome data to drive behaviour change*

Publish costs and outcomes for each hospital and health services on Queensland Health website (publicly available)

Use International Consortium for Health Outcomes Measurement or other standard set of outcome measures to report health outcomes and longer‐term outcomes (eg quality of life)

Report variation in CS rates between clinicians, between public and private

**3. Workforce capability**

*Develop evidence‐informed Queensland Health guidelines that facilitate clinical judgement and women's choices*

Audit clinicians' access to, and application of, clinical guidelines

Develop procedures that embed guidelines in practice

Integrate other policies with normal birth guideline to avoid ‘clashing’

*Facilitate women's informed decision‐making about interventions*

Educate workforce about how to critique the evidence; and how to normalise, individualise, and counsel women about the risks and benefits of medical interventions

Use ‘real world, real time evidence’ to create risk predictive models

*Educate workforce to promote normal birth and address fear*

Deliver targeted, multi‐disciplinary education forums about the benefits of normal birth and how to facilitate it

Facilitate dialogue between Health and Human Services lawyers and obstetricians to de‐escalate fears of malpractice litigation

Identify normal birth clinical leaders and champions who can expose clinicians and managers to normal birth

Educate workforce and consumers to use a ‘speak up for safety’ process to advocate for normal birth

*Build workforce capacity and team function*

Provide clinical‐peer supervision model for midwives

Provide advocacy training to help midwives ‘keep birth normal’

Provide a program (eg ALICE (a woman‐centred care program offered by Queensland Health for clinicians to further develop collaborative leadership) program) that enhances multi‐disciplinary team functioning

Revise human resources practices that undermine sustainability of midwifery models in rural areas (eg one‐shift cannot be undertaken while on maternity leave)

### Workshop 2

Prior to the workshop, participants were emailed a survey link inviting them to prioritise activities (full list in Box 2) into the three priority areas for potential inclusion in the Strategy using the MoSCoW method. This approach asks participants to prioritise which activities would provide the best return on investment using the following four categories: Must‐do; Should‐do; Could‐do; Will not do. Objectives of the workshop were to:

- develop consensus on short, mid, or long‐term activities as part of the Strategy
- rate the degree of complexity of each activity – consider enabling and resisting factors
- rate the degree of difficulty of each activity – consider enabling and resisting factors
- consider creating a collaborative as an implementation approach, and
- consider/design elements of the full Strategy.

Most participants responded to the priorities survey (76%). The survey results were presented and discussed at the workshop, to move toward consensus on activities to form part of the Strategy. Discussion included consideration of degree of complexity and difficulty of activities, and whether certain activities could be integrated or embedded into a larger system change.

### Workshop 3

Prior to the workshop, participants were emailed the draft Strategy to review and formulate feedback at the final workshop. Workshop objectives were to revise and agree on the Strategy. Participants decided that the Strategy should focus more broadly on ‘normal birth’ as opposed to just on CS. Normal birth was defined as the normalisation of the physiological process that is birth, as opposed to being focused on a type of birth (vaginal, operative vaginal, CS) or level of medical intervention. Participants discussed the development of a Normal Birth Collaborative as a vehicle to promote, embed and sustain the recommendations. Discussion included purpose and remit, membership, and initial priorities of the Collaborative.

## THE STRATEGY

Participants agreed that the following principles should underpin development of the Strategy:

- change culture to promote normal birth and mitigate fear of birth
- centre women's informed decision‐making, access, and control
- respect the scope of practice of midwives, obstetricians, and other maternity care providers
- use cost‐effective, evidence‐based solutions, and
- privilege consumer voices in service design and delivery.

Using these principles, we developed the Strategy to promote normal birth which has five^5^ components.

1. Universal access to midwifery continuity of carer and choice of place of birth. Redesign maternity care so that every woman has the *opportunity* to receive midwifery care from a known midwife during pregnancy, birth and the first 6 weeks afterward. This model of care includes obstetric consultation, collaboration and referral as indicated.
2. Multi‐disciplinary normal birth education: deliver multi‐disciplinary education for maternity staff that includes obstetricians, midwives, midwifery and medical students, and maternity consumers.
3. Co‐designed resources for obstetricians and midwives to facilitate informed decision‐making: develop, implement, and evaluate a resource package to facilitate women's informed decision‐making in areas that directly, or indirectly, impact normal birth.
4. Initiatives to embed respectful maternity care and positive workplace culture: facilitate midwives, obstetricians, and maternity leaders to deliver high‐value, quality maternity care.
5. Establishment of a sustainable Normal Birth Collaborative: establish and sustain a multi‐disciplinary collaborative of maternity leaders, obstetricians, midwives, maternity consumers, and maternity researchers to drive the Strategy and champion normal birth throughout Queensland with a view to becoming a national leader with global influence.

Each component bundles together several evidence‐based activities. The full details of each component will be published in the forthcoming full report.

## Funding

This work was commissioned and funded by Clinical Excellence Queensland, Queensland Health.
